# Texas State Medical Association

**Published:** 1893-06

**Authors:** H. A. West

**Affiliations:** Galveston, Texas


### Society Notes.

### TEXAS STATE MEDICAL ASSOCIATION.

### Notes on the Recent Meeting in Galveston.

GALVESTON, TEXAS, May 29, 1893.

*Editor Daniel's Texas Medical Journal:*

There are some points of interest concerning the recent meeting of the Association which were not mentioned in the epitome published in your May number, and which doubtless may be entertaining to some of your readers. In regard to the attendance — the register shows about one hundred and sixty-five members present, including thirty-seven new members.—This may look like a small proportion, but considering that Galveston is upon

*In debate at Galveston our facetious friend, Dr. Q. C. Smith alluded to Prof. Keiller "as the gentleman from the land o' cakes and ale."

the extreme southern border of the State, the attendance was fair, about fifty more registering than at Tyler. The program which was issued some weeks in advance of the meeting, must have been a revelation to those who had dismally predicted that the Association was fast approaching a condition of "innocuous disuetude."—It presented a list of about fifty-four papers, which, in point of ability as the Transactions will show, favorably compares with those of any similar body in America. This result was due to the careful selection of officers of Sections at Tyler. Unfortunately time was wanting to read more than half of the papers, which demonstrates the necessity hereafter of dividing the work into Sections, or, at least, of having the Sections of General Medicine, Surgery and Gynecology in the general session, and the other Sections separately. At Tyler there was a woeful lack of discussion—it was truly a Quaker meeting. The Transactions of this year will show an intelligent and interesting discussion of almost every paper read. It is hardly necessary to state that this happy result was due to the timely announcement of the program.

CHANGES IN THE CONSTITUTION.

A radical change was made in the first article,—the bars have been let down,—our Association is no longer to be composed of men only; we have opened our arms to receive our sisters in the profession. The ommittee upon revision had not thought of or suggested the change, it was a spontaneous and unpremeditated act of nineteenth century progress and enlightenment exemplified by the members present. A vital defect existed in the old constitution, whereby process against offenders could only originate in county societies. This is remedied. While every safeguard is provided to make trials equitable, the Association now has jurisdiction and can purge itself when necessary, of objectionable members without appeal. A matter upon which there was manifested considerable difference of opinion, was the abrogation of the ordinance prohibiting publication of papers prior to their appearance in the Transactions. I think this was a wise and progressive enactment. Authors of original and valuable papers do not want them buried in the Transactions; some might be willing to sacrifice themselves for the love they bear the Association, but the majority are not; the only compensation they ask is, that their contributions may be offered to the world through a more widely circulating medium. This privilege is allowed by some of the foremost medical organizations in the world, and I have no doubt but that it will prove the incentive to the production of a better class of papers for ours.

Of the social features of the occasion it would not be becoming in the writer to speak in detail, but one thing I wish to mention: that is, what appears to me as the charming success of substituting the buffet banquet and concert, where ladies could grace the occasion by their presence and smiles, for the traditional drinking bout and maudlin speech making, ordinarily called a banquet, which has heretofore prevailed. It was mainly to try this experiment that induced me to accept the responsibility and labor incident to the chairmanship of the Committee of Arrangement. The general impression made upon my mind was that the recent meeting was a grand success in every way, and a most favorable augury for the future progress and usefulness of our beloved old Association. H. A. WEST, M. D.

